# Fully automated quantification of biventricular volumes and function in cardiovascular magnetic resonance: applicability to clinical routine settings

**DOI:** 10.1186/s12968-019-0532-9

**Published:** 2019-04-25

**Authors:** Sören J. Backhaus, Wieland Staab, Michael Steinmetz, Christian O. Ritter, Joachim Lotz, Gerd Hasenfuß, Andreas Schuster, Johannes T. Kowallick

**Affiliations:** 1 Department of Cardiology and Pneumology, University Medical Center Göttingen, Georg-August University, Göttingen, Germany; 2 German Center for Cardiovascular Research (DZHK), Partner Site Göttingen, Göttingen, Germany; 3 Institute for Diagnostic and Interventional Radiology, University Medical Centre Göttingen, Georg-August University, Robert-Koch-Str. 40, 37075 Göttingen, Germany; 4 Department of Pediatric Cardiology and Intensive Care Medicine, University Medical Center Göttingen, Georg-August University, Göttingen, Germany; 5 Department of Cardiology, Royal North Shore Hospital, The Kolling Institute, Nothern Clinical School, University of Sydney, Sydney, Australia

## Abstract

**Background:**

Cardiovascular magnetic resonance (CMR) represents the clinical gold standard for the assessment of biventricular morphology and function. Since manual post-processing is time-consuming and prone to observer variability, efforts have been directed towards automated volumetric quantification. In this study, we sought to validate the accuracy of a novel approach providing fully automated quantification of biventricular volumes and function in a “real-world” clinical setting.

**Methods:**

Three-hundred CMR examinations were randomly selected from the local data base. Fully automated quantification of left ventricular (LV) mass, LV and right ventricular (RV) end-diastolic and end-systolic volumes (EDV/ESV), stroke volume (SV) and ejection fraction (EF) were performed overnight using commercially available software (suiteHEART®, Neosoft, Pewaukee, Wisconsin, USA). Parameters were compared to manual assessments (QMass®, Medis Medical Imaging Systems, Leiden, Netherlands). Sub-group analyses were further performed according to image quality, scanner field strength, the presence of implanted aortic valves and repaired Tetralogy of Fallot (ToF).

**Results:**

Biventricular automated segmentation was feasible in all 300 cases. Overall agreement between fully automated and manually derived LV parameters was good (LV-EF: intra-class correlation coefficient [ICC] 0.95; bias − 2.5% [SD 5.9%]), whilst RV agreement was lower (RV-EF: ICC 0.72; bias 5.8% [SD 9.6%]). Lowest agreement was observed in case of severely altered anatomy, e.g. marked RV dilation but normal LV dimensions in repaired ToF (LV parameters ICC 0.73–0.91; RV parameters ICC 0.41–0.94) and/or reduced image quality (LV parameters ICC 0.86–0.95; RV parameters ICC 0.56–0.91), which was more common on 3.0 T than on 1.5 T.

**Conclusions:**

Fully automated assessments of biventricular morphology and function is robust and accurate in a clinical routine setting with good image quality and can be performed without any user interaction. However, in case of demanding anatomy (e.g. repaired ToF, severe LV hypertrophy) or reduced image quality, quality check and manual re-contouring are still required.

**Electronic supplementary material:**

The online version of this article (10.1186/s12968-019-0532-9) contains supplementary material, which is available to authorized users.

## Introduction

Cardiovascular magnetic resonance (CMR) imaging is the gold standard for the assessment of cardiac function and morphology [1, 2]. Left ventricular (LV) ejection fraction (EF) is the most established parameter for cardiac functional assessments in clinical routine and is used for the evaluation of disease severity, treatment follow-up and risk assessment for adverse events [3, 4].

To extract clinically relevant information such as LV mass, LV and right ventricular (RV) end-diastolic and end-systolic volume (EDV/ESV), stroke volume (SV) and EF, accurate post-processing of the cine CMR images is required. In daily clinical routine, post-processing is typically performed manually by delineating endocardial and epicardial LV borders as well as endocardial RV borders in all short-axis (SAX) slices covering the ventricles from atrioventricular ring to apex, in both end-diastolic and end-systolic phases. This task is time-consuming, tedious and subject to observer-variability [2, 5–7]. Emerging post-processing software based on deep-learning algorithms using convolutional neural networks now offer a fully automated approach for LV and RV volume assessments and have recently become commercially available [8]. Initial evaluations of these automated approaches are promising [9], however often based on pre-selected cases with excellent image quality or ‘cropped’ data [9, 10], i.e. SAX stacks are manually triaged to only include end-diastolic and end-systolic images effectively covering the ventricles before applying the automatic algorithm. Importantly, most of the observer-variability results from discrepancies in defining the most apical and basal short-axis SAX slices [11], which is whitewashed by previous manual ‘cropping’ of SAX stacks.

At the current time, it remains unknown whether fully automated quantification of biventricular morphology and function is feasible and accurate in clinical CMR routine. Accordingly, the aim of the present study was to evaluate the feasibility and accuracy of fully automated biventricular assessment of morphology and function in a variety of CMR data (neither pre-selected nor pre-processed) taken from a real-world data base of a tertiary care CMR unit.

## Methods

### Study design

The study population consisted of 300 randomly selected patients referred to CMR within clinical routine care between 2016 and 2018. The CMR imaging protocol was employed on clinical 1.5 or 3 Tesla (Magnetom Symphony or Magnetom Skyra, Siemens Healthineers, Erlangen, Germany) CMR scanners. Protocols were employed as appropriate for clinical routine, all of which including electrocardiogram (ECG)-gated balanced steady-state free precession (bSSFP) cine sequences for a SAX stack. Typical imaging parameters were as follows: 25 frames/cardiac cycle, pixel spacing 0.8 mm × 0.8 mm, 8 mm slice thickness as well as inter-slice gap, TE 1.5 ms, TR 3 ms. The study was approved by the Ethics Committee of the University Hospital Goettingen and complied with the Declaration of Helsinki. The Ethics Committee gave permission to waive informed consent for this retrospective analysis. Furthermore, agreement was assessed between the fully automated algorithm and expert consensus contours based on the Society for Cardiovascular Magnetic Resonance (SCMR) consensus data consisting of 15 cases with different pathologies [12].

### CMR analyses

Volumetric analyses were performed manually in short-axis orientations by an experienced investigator according to standardized recommendations [11] using commercially available post-processing software (QMass®, Version 3.1.16.0, Medis Medical Imaging Systems, Leiden, Netherlands). Fully automated segmentation was performed employing dedicated commercially available software (suiteHEART®, Version 4.0.6, Neosoft, Pewaukee, Wisconsin, USA). The papillary muscles were included within the myocardium, trabecular tissue was excluded from the myocardial mass using both, the manual (QMass®) and the automated (suiteHEART®) software. Manual segmentation was performed by simple delineation of the LV endocardial- and epicardial borders and the RV endocardial border with Bézier smoothing at end-diastole and end-systole. No thresholding or edge detection was applied. Cross-referencing from long-axis locations was used to adjust for systolic atrioventricular ring descent. Fully automated segmentation was done overnight without any user-interaction neither by pre-processing the acquired short axis stack nor by post-processing automatically traced borders. Analyses included LV mass, and biventricular EDV, ESV, SV and EF. Agreement was tested between manual and fully automated analyses. Reproducibility was tested by reapplying the fully automated tracking algorithm on 20 randomly selected patients and by manual volumetric analyses by two experienced investigators including intra- and inter-observer reproducibility. All operators were blinded to each other’s results. Furthermore, the analysis time needed to perform manual segmentations was measured in the subset of 20 patients. The presence and relevance of artefacts impacting image quality was graded adopting the criteria proposed by Klinke et al. [13] taking wrap around, respiratory ghost, cardiac ghost, image blurring, metal and shimming artefacts into account **(**Table 1**)**. One point was given if the artefact impeded the visualization of > 1/3 of the ventricular endocardial border at end-systole and/or end-diastole on a single SAX slice. If such artefact involved 2 slices or ≥ 3 slices, 2 and 3 points were given, respectively. Furthermore, accurate short-axis orientation was evaluated, resulting in an image quality score between 0 (= excellent quality) and 6 (= poor quality). Image quality scores were separately assessed for the LV and RV myocardium.

**Table 1:** Quality assessment of cine short-axis (SAX) images. The image quality score corresponds to the sum of qualitative scoring based on 6 criteria (range of score: 0–5). One point was given if an artefact impeded the visualization of > 1/3 of the ventricular endocardial border at end-systole and/or end-diastole on a single SAX slice. If such artefact involved 2 slices or ≥ 3 slices, 2 and 3 points were given, respectively. Incorrect short-axis orientation was graded with 2 points

	0	1	2	3	Maximum Score
1. Wrap around	No	1 slice	2 slices	≥3 slices	3
2. Respiratory ghost	No	1 slice	2 slices	≥3 slices	
3. Cardiac ghost	No	1 slice	2 slices	≥3 slices	
4. Image blurring / mis-triggering	No	1 slice	2 slices	≥3 slices	
5. Metallic artefacts	No	1 slice	2 slices	≥3 slices	
6. Orientation of stack	Correct	–	Incorrect	–	2

For the SCMR consensus data, only LV parameters were compared between automated analyses and manual expert consensus parameters, since RV parameters were not provided. According to the method described by Suinesiaputra et al. [12], papillary muscles and trabecular tissue were excluded from the myocardial mass.

### Statistics

Continuous variables were checked for normal distribution using the Shapiro-Wilk test and are presented as median with interquartile range (IQR). Biventricular volumes and LV mass were indexed to body surface area. Dependent variables were tested using the Wilcoxon signed-rank test. Agreement of manual and automated analyses as well as intra- and inter-observer variability was assessed first using Bland-Altman analysis [mean difference between measurements with 95% confidence interval (CI)] [14], second intra-class correlation coefficients (ICC) based on a model of absolute agreement, considered excellent if ICC > 0.74, good between 0.60 and 0.74, fair between 0.4 and 0.59 and poor below 0.4 [15], and third the coefficient of variation (CoV, = standard deviation [SD] of the differences divided by the mean) [16, 17]. *P*-values provided are two-sided, an alpha level below 0.05 was considered statistically significant. Statistical analyses were performed using IBM SPSS Statistic Software Version 24 (International Business Machines, Armonk, New York, USA) and Microsoft Excel (Microsoft, Redmond, Washington, USA).

## Results

### Study population

Patient characteristics and cardiac volumes for both manual and automated assessments are presented in Table 2. Biventricular automatic segmentation was feasible in all 300 cases. In comparison with manual evaluations, automatic assessments depicted higher LV volumes, lower LVEF, higher LV mass as well as higher RV EDV, RV SV and RVEF (*p* < 0.001 for all). The study population consisted of 100 referrals to evaluate ischemic heart disease, 120 patients with myocardial disease, 70 patients with congenital heart disease and 10 others. Table 3 provides an overview of clinical indications. There were 31 patients imaged after aortic valve replacement (AVR) of whom 18 received transcatheter aortic valve replacement (Edwards SAPIEN 3™, Edwards Lifesciences, Irvine, California, USA), 7 patients after open-surgery AVR using a bioprosthesis (Carpentier-Edwards Perimount™, Edwards Lifesciences) and 6 patients after open-surgery AVR with a mechanical aortic valve (SJM Regent™, St. Jude Medical Inc., St Paul, Minnesota, USA).

**Table 2:** Demographics and biventricular volumes

Parameter	Study population		
Gender (f/m)	118/182		
Age	52 (30, 71)		
BSA	1.92 (1.72, 2.05)		
	Automated	Manual	p
LV Mass, g/m^2^	59.8 (48.8, 74.1)	58.0 (46.0, 73.0)	**< 0.001**
LV EDV, ml/m^2^	88.5 (77.4, 105.8)	83.0 (71.0, 100.0)	**< 0.001**
LV ESV, ml/m^2^	37.7 (29.4, 51.1)	33.0 (25.0, 47.0)	**< 0.001**
LV SV, ml/m^2^	48.8 (41.2, 55.8)	48.0 (40.0, 56.0)	0.133
LV EF	58.0 (48.0, 63.0)	60.0 (51.0, 66.0)	**< 0.001**
RV EDV, ml/m^2^	87.0 (71.1, 107.4)	79.0 (65.0, 97.0)	**< 0.001**
RV ESV, ml/m^2^	38.4 (26.9, 48.0)	39.0 (28.0, 51.0)	0.125
RV SV, ml/m^2^	47.8 (40.3, 55.9)	40.5 (32.0, 48.0)	**< 0.001**
RV EF	56.0 (50.0, 63.0)	51.0 (44.0, 58.0)	**< 0.001**

Continuous variables are expressed as median and interquartile range (IQR) and were tested using the Wilcoxon signed-rank test. *LV/RV* left/right ventricle, *EDV/ESV* end-diastolic/systolic volume, *SV* stroke volume, *EF* ejection fraction

Numbers in bold type indicate a significant difference

**Table 3:** Clinical CMR indications

	Number of patients
Ischemic Heart Disease	100
Coronary Heart Disease	97
Acute Myocardial Infarction	3
Myocardial disease	120
Myocarditis	64
Arrhythmogenic RV Cardiomyopathy	19
Dilated Cardiomyopathy	14
Hypertrophic Cardiomyopathy	11
Sarcoidosis	7
Iron Overload Cardiomyopathy	3
Non-Compaction Cardiomyopathy	1
Anderson Fabry Disease	1
Congenital Heart Disease	70
Repaired Tetralogy of Fallot	47
Aortic Coarctation	12
Atrial Septal Defect	6
Aortic Dilatation in Bicuspid Aortic Valve	5
Others	10
Cardiac Mass	5
Rheumatic Disease	2
Pericarditis Constrictiva	1
Pericardial Effusion	1
Pulmonary Arterial Hypertension	1

*RV* right ventricular

### Image quality and post-processing

**Fig. 1:**
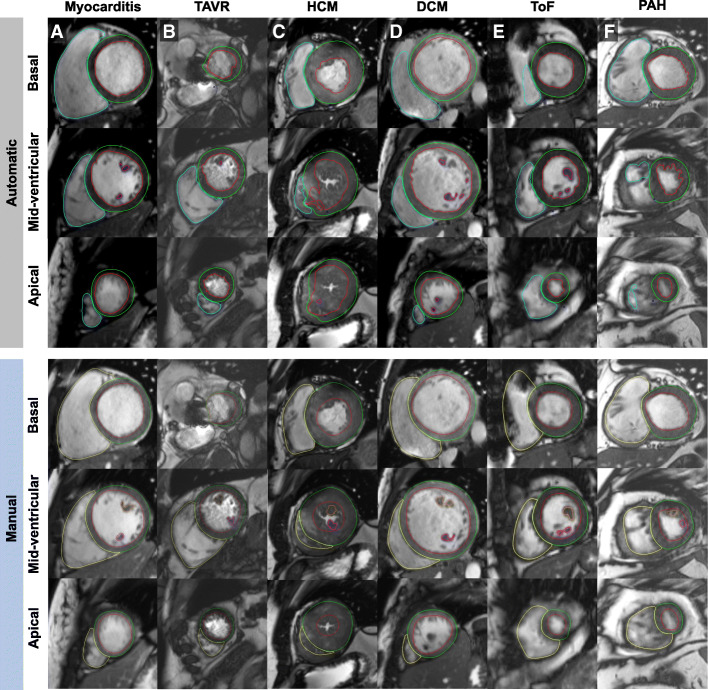
Fully automated biventricular segmentation (upper panel) and manual segmentation (lower panel) of 6 representative cases. The figure comprises examples with good automated segmentation results (**a**-**b**) and limited automated segmentation results (**c**-**f**). Segmentation results of all phases and all short-axis slices can be found in the supplementary material. **a** End-diastolic phases of a patient with suspected myocarditis (1.5 T) and excellent biventricular segmentation. **b** End-systolic phases of a patient after transcatheter aortic valve replacement (TAVR) imaged at 3 T showing good segmentation results, except for insufficient segmentation of papillary muscles. **c** End-systolic phases of a patient with severe hypertrophic cardiomyopathy (HCM) imaged at 1.5 T with low accuracy of biventricular segmentations. **d** End-diastolic phases of a patient with dilated cardiomyopathy (DCM) imaged at 1.5 T with underestimation of right ventricular (RV) volume at the basal level. Also note misinterpretation of two apical thrombi as papillary muscles. **e** End-systolic phases of a patient with repaired Tetralogy of Fallot (ToF) with underestimation of RV volume due to severe metallic artefacts caused by sternal wires. **f** End-systolic phases of a patient with pulmonary arterial hypertension (PAH) with underestimation of RV volume most likely due to RV hypertrabeculation

LV-image quality was graded with 1.0 (SD 1.3) (Score 0 *n* = 168, Score 1 *n* = 19, Score 2 *n* = 46, and Score 3 *n* = 67 points. RV-image quality was graded with 1.1 (SD 1.3) (Score 0 *n* = 151, Score 1 *n* = 37, Score 2 *n* = 39, and Score 3 *n* = 73 points. Appropriate short-axis orientation was fulfilled in 298 case, the highest image quality score assigned was 3. Manual post-processing took on average 11.3 ± 1.5 min as opposed to automated pre-processing with < 1 min/SAX stack. Representative examples of high and low segmentation accuracy are given in Fig. 1**.** Corresponding videos including automatic segmentation of all phases and SAX slices can be found in Additional file 1.

### Agreement of manual and automated analyses

**Fig. 2:**
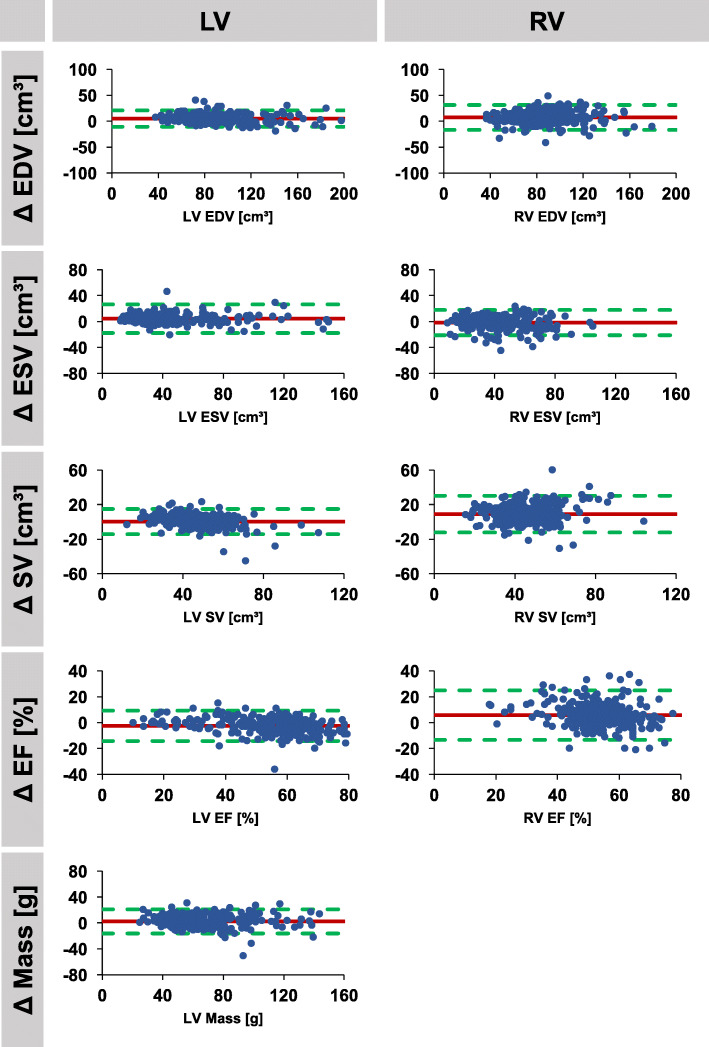
Agreement of automatically and manually derived biventricular morphology and function. Bland Altman plots (automatic – manual) are shown for the entire study collective (*n* = 300). LV/RV: left/right ventricle, EDV/ESV: end-diastolic/systolic volume, SV: stroke volume, EF: ejection fraction, Δ: difference

**Fig. 3:**
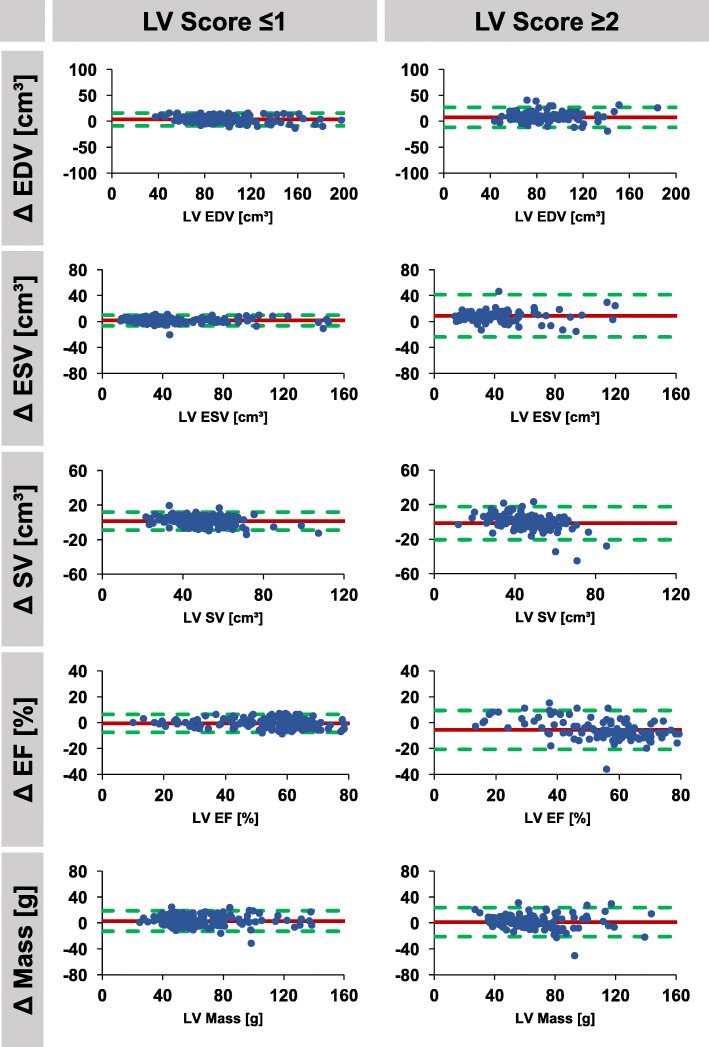
Agreement of automatically and manually derived left ventricular parameters according to image quality. Bland Altman plots (automatic – manual) are shown for studies with good image quality (score ≤ 1, *n* = 187) and for studies with reduced image quality (score ≥ 2, *n* = 113). LV: left ventricle, EDV/ESV: end-diastolic/systolic volume, SV: stroke volume, EF: ejection fraction, Δ: difference

**Fig. 4:**
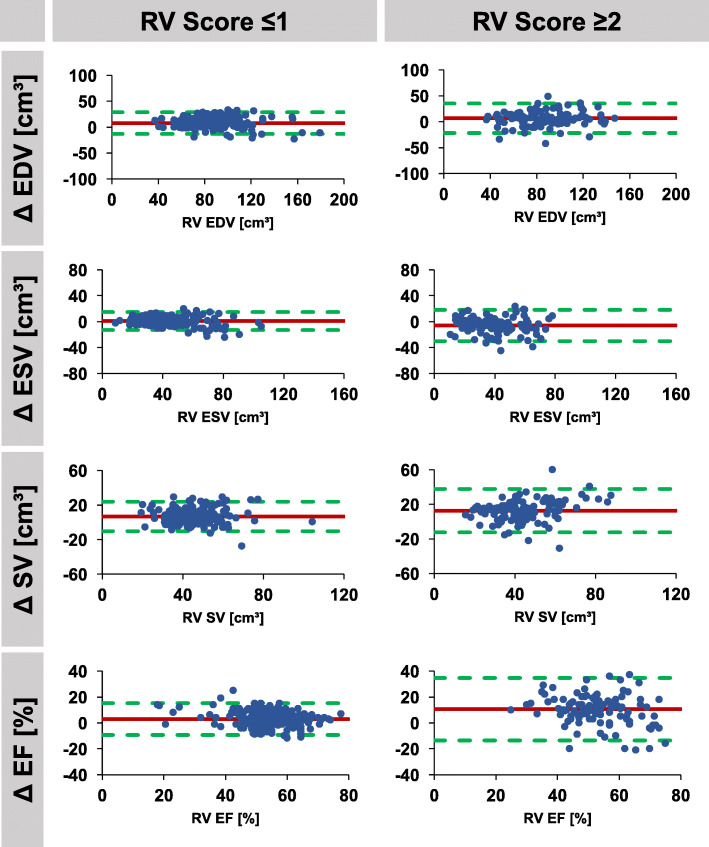
Agreement of automatically and manually derived right ventricular parameters according to image quality. Bland Altman plots (automatic – manual) are shown for studies with good image quality (score ≤ 1, *n* = 188) and for studies with reduced image quality (score ≥ 2, *n* = 112). RV: right ventricle, EDV/ESV: end-diastolic/systolic volume, SV: stroke volume, EF: ejection fraction, Δ: difference

Results comparing automated and manual volume assessments including mean differences with corresponding SD, ICC and CoV are presented in Tables 4 and 5. Corresponding Bland-Altman plots are presented in Figs. 2, 3 and 4. Agreement of manual and automated assessments in the overall cohort of 300 patients was excellent for all LV parameters (ICC ≥0.91), best for EDV (ICC 0.98) closely followed by ESV (ICC 0.96) as well as mass and EF (both ICC 0.95). The automated algorithm slightly overestimated LV mass, EDV and ESV while underestimating LV EF (mean difference − 2.5%, limits of agreement [LOA] -14.6 to 9.1%), *p* < 0.001). Agreement for RV volumes was excellent for RV EDV and ESV (both ICC 0.92) and good for RV SV (ICC 0.73) and EF (ICC 0.72). Similar to LV measurement, the automatic algorithm overestimated RV EDV, and also RV EF (mean difference 5.8%, LOA -13.0 to 24.6%, p < 0.001). Higher field strength (3 vs 1.5 Tesla) was associated with reduced agreement in biventricular volumes, though it was also associated with a decrease in image quality (1.5 T: LV image quality score 0.8 (SD 1.2), RV image quality score 0.7 (SD 1.1); 3.0 T: LV image quality score 1.4 (SD 1.3), RV image quality score 1.1 (SD 1.3); p < 0.001 for all). Similarly, aortic valve replacement resulted in lower agreement but was also accompanied by lower image quality (LV image quality score 1.9 (SD 1.2); RV image quality score 2.0 (SD 1.2). Repaired ToF was associated with decreased RV image quality (RV image quality score 1.8 [SD 1.1]) but preserved LV image quality (LV image quality score 0.62 [0.99]). Despite preserved LV image quality, agreement was reduced for both LV and RV volumes (Table 3**.**).

**Table 4:** Agreement between manual and automated segmentations. Agreement was analysed in the entire study group (*n* = 300) as well as in subgroups according to field strength, aortic valve replacement and repaired Tetralogy of Fallot

	Parameter	Mean Difference (SD of the Diff.)	ICC (95% CI)	CoV (%)
All	LV Mass	2.4 (9.3)	0.95 (0.94–0.97)	14.6
(*n* = 300)	LV EDV	5.0 (7.9)	0.98 (0.94–0.99)	8.5
	LV ESV	4.4 (11.1)	0.96 (0.94–0.97)	25.0
	LV SV	0.3 (7.3)	0.91 (0.89–0.93)	15.1
	LV EF	−2.5 (5.9)	0.95 (0.92–0.97)	10.6
	RV EDV	7.4 (12.0)	0.92 (0.81–0.96)	14.0
	RV ESV	−1.6 (9.8)	0.92 (0.89–0.93)	24.0
	RV SV	9.0 (10.6)	0.73 (0.26–0.87)	23.5
	RV EF	5.8 (9.6)	0.72 (0.47–0.83)	17.8
1.5 T	LV Mass	4.0 (7.2)	0.96 (0.91–0.98)	11.7
(*n* = 132)	LV EDV	4.4 (8.4)	0.98 (0.96–0.99)	8.8
	LV ESV	2.9 (6.1)	0.99 (0.98–1.00)	12.2
	LV SV	1.1 (6.4)	0.95 (0.92–0.96)	13.7
	LV EF	−1.5 (4.9)	0.97 (0.96–0.98)	9.3
	RV EDV	10.6 (9.6)	0.90 (0.33–0.97)	11.7
	RV ESV	2.1 (8.0)	0.94 (0.91–0.96)	20.5
	RV SV	8.5 (8.8)	0.72 (0.11–0.88)	20.6
	RV EF	3.9 (8.8)	0.77 (0.62–0.86)	16.5
3 T	LV Mass	0.1 (9.7)	0.97 (0.95–0.98)	13.2
(*n* = 90)	LV EDV	5.5 (7.3)	0.98 (0.91–0.99)	7.8
	LV ESV	6.9 (17.7)	0.88 (0.79–0.92)	40.5
	LV SV	−1.4 (8.5)	0.84 (0.75–0.89)	17.1
	LV EF	−3.8 (6.9)	0.92 (0.83–0.96)	12.3
	RV EDV	5.8 (12.2)	0.89 (0.79–0.94)	15.0
	RV ESV	−0.7 (7.9)	0.94 (0.92–0.96)	20.7
	RV SV	6.2 (11.0)	0.64 (0.35–0.79)	25.3
	RV EF	4.1 (8.4)	0.83 (0.68–0.90)	15.3
Aortic Valve replacement	LV Mass	1.3 (11.5)	0.89 (0.76–0.94)	16.7
(*n* = 31)	LV EDV	5.0 (7.7)	0.97 (0.91–0.99)	8.7
	LV ESV	5.5 (5.6)	0.97 (0.76–0.99)	14.8
	LV SV	−0.4 (7.6)	0.93 (0.85–0.96)	15.2
	LV EF	−4.4 (6.4)	0.92 (0.73–0.97)	10.8
	RV EDV	6.0 (13.9)	0.90 (0.78–0.95)	16.6
	RV ESV	−6.9 (10.6)	0.86 (0.60–0.94)	28.9
	RV SV	12.8 (15.0)	0.54 (0.00–0.79)	31.6
	RV EF	10.3 (13.7)	0.54 (0.00–0.79)	23.7
Tetralogy of Fallot	LV Mass	2.9 (11.3)	0.75 (0.55–0.86)	23.0
(*n* = 47)	LV EDV	5.6 (7.8)	0.91 (0.69–0.96)	9.4
	LV ESV	3.1 (6.4)	0.83 (0.66–0.91)	18.7
	LV SV	1.9 (6.5)	0.87 (0.76–0.93)	13.1
	LV EF	−1.5 (5.3)	0.73 (0.52–0.85)	8.9
	RV EDV	2.6 (14.3)	0.94 (0.90–0.97)	13.5
	RV ESV	− 10.1 (10.5)	0.82 (0.25–0.93)	20.2
	RV SV	13.0 (9.4)	0.81 (0.00–0.94)	17.4
	RV EF	11.3 (7.5)	0.41 (0.00–0.73)	14.8

Biventricular volumes and LV mass were indexed to body surface area. T: Tesla. *SD* standard deviation, *ICC* intraclass correlation coefficient, *CoV* coefficient of variation, *LV* left ventricular, *RV* right ventricular, *EDV/ESV* end-diastolic/systolic volume, *SV* stroke volume, *EF* ejection fraction

**Table 5:** Agreement between manual and automated analyses according to image quality

		Parameter	Mean Difference (SD of the Diff.)	ICC (95% CI)	CoV (%)
Good image quality (Score ≤ 1)	LV (*n* = 187)	LV Mass	3.0 (7.9)	0.96 (0.94–0.98)	12.8
		LV EDV	3.4 (6.2)	0.99 (0.97–0.99)	6.5
		LV ESV	1.7 (4.1)	0.99 (0.99–1.00)	9.0
		LV SV	1.5 (5.2)	0.95 (0.93–0.96)	10.5
		LV EF	−0.6 (3.5)	0.98 (0.98–0.99)	6.2
	RV (*n* = 188)	RV EDV	7.8 (10.5)	0.93 (0.75–0.97)	12.2
		RV ESV	1.0 (6.9)	0.96 (0.95–0.97)	16.9
		RV SV	6.7 (8.5)	0.79 (0.40–0.90)	18.9
		RV EF	3.0 (6.1)	0.88 (0.78–0.93)	11.5
Reduced image quality (Score ≥ 2)	LV (*n* = 113)	LV Mass	1.3 (11.2)	0.94 (0.91–0.96)	16.7
		LV EDV	7.5 (9.7)	0.95 (0.82–0.98)	10.8
		LV ESV	8.9 (16.3)	0.87 (0.74–0.93)	37.9
		LV SV	−1.6 (9.6)	0.86 (0.80–0.90)	20.5
		LV EF	−5.6 (7.5)	0.90 (0.67–0.96)	13.8
	RV (*n* = 112)	RV EDV	6.7 (14.3)	0.91 (0.83–0.95)	16.8
		RV ESV	−5.9 (12.1)	0.84 (0.70–0.90)	30.2
		RV SV	12.7 (12.6)	0.67 (0.02–0.86)	27.9
		RV EF	10.6 (12.1)	0.56 (0.03–0.77)	22.5

Biventricular volumes and LV mass were indexed to body surface area. *SD* standard deviation, *ICC* intraclass correlation coefficient, *CoV* coefficient of variation, *LV* left ventricular, *RV* right ventricular, *EDV/ESV* end-diastolic/systolic volume, *SV* stroke volume, *EF* ejection fraction

If classified according to image quality score, 0 to 1 point was associated with considerable better agreement than 2 to 3 points, both for LV and RV automated analyses. Considering an image quality score of ≤1, both LV and RV agreements were excellent for all variables with a bias of − 0.6% (LOA -7.6 to 6.4%) and 3.0% (LOA -9.2 to 15.2%) for LV EF and RV EF, respectively. However, large differences were observed in case of reduced image quality (score ≥ 2) with a bias of − 5.6% (LOA -20.6 to 9.4%) and 10.6% (LOA -13.6 to 34.8%) for LV EF and RV EF, respectively. LV and RV stroke volumes were very consistent in automated analyses, LV 48.1 ml/m^2^ compared to RV 47.6 ml/m^2^ in median, *p* = 0.435.

Results from the comparison between automatically and manually derived expert consensus LV parameters based on the SCMR consensus data are provided in the Additional file 1. In accordance with the study’s results, agreement was excellent in the majority of cases (ICC ≥ 0.95 for all LV parameters) (Additional file 2: Tables S1 and S2). There was one patient with a 20% difference in LV EF between automatic and manual expert results (Case # 15), which was a patient with severe LV hypertrophy (Additional file 2: Table S1, Figure S1), similar to the case shown in Fig. 1c.

### Reproducibility

Reproducibility for manual segmentations was better for LV than for RV measurements. The automated algorithm yielded exactly the same results when being reapplied. Table 6 shows ICC, CoV and mean differences (SD) within and between observers.

**Table 6:** Reproducibility of manual and automated analyses

		Parameter	Mean Difference (SD of the Diff.)	ICC (95% CI)	CoV (%)
Manual	Intra-observer	LV Mass	−0.3 (3.4)	0.99 (0.97–1.00)	5.3
		LV EDV	−3.3 (3.4)	0.99 (0.91–1.00)	4.0
		LV ESV	−2.6 (3.0)	0.99 (0.95–1.00)	8.4
		LV SV	−0.6 (3.9)	0.97 (0.93–0.99)	8.0
		LV EF	1.7 (3.7)	0.98 (0.94–0.99)	6.3
		RV EDV	−1.5 (6.7)	0.96 (0.90–0.98)	9.6
		RV ESV	1.8 (4.3)	0.95 (0.87–0.98)	14.2
		RV SV	−3.5 (6.3)	0.81 (0.49–0.93)	15.6
		RV EF	−2.9 (5.1)	0.82 (0.49–0.93)	8.9
	Inter-observer	LV Mass	−0.7 (3.3)	0.99 (0.97–1.00)	5.6
		LV EDV	−5.6 (5.8)	0.97 (0.74–0.99)	6.7
		LV ESV	−6.3 (4.8)	0.97 (0.46–0.99)	12.5
		LV SV	0.8 (3.7)	0.97 (0.93–0.99)	7.7
		LV EF	5.2 (57.9)	0.94 (0.44–0.98)	8.0
		RV EDV	−15.5 (7.8)	0.82 (0.00–0.96)	10.0
		RV ESV	−7.3 (5.7)	0.84 (0.00–0.96)	16.4
		RV SV	−8.1 (6.9)	0.75 (0.00–0.93)	16.2
		RV EF	1.3 (7.2)	0.62 (0.04–0.85)	12.9
Automatic	Intra-observer	LV Mass	0.0 (0.0)	1.00	0.0
		LV EDV	0.0 (0.0)	1.00	0.0
		LV ESV	0.0 (0.0)	1.00	0.0
		LV SV	0.0 (0.0)	1.00	0.0
		LV EF	0.0 (0.0)	1.00	0.0
		RV EDV	0.0 (0.0)	1.00	0.0
		RV ESV	0.0 (0.0)	1.00	0.0
		RV SV	0.0 (0.0)	1.00	0.0
		RV EF	0.0 (0.0)	1.00	0.0
	Inter-observer	LV Mass	0.0 (0.0)	1.00	0.0
		LV EDV	0.0 (0.0)	1.00	0.0
		LV ESV	0.0 (0.0)	1.00	0.0
		LV SV	0.0 (0.0)	1.00	0.0
		LV EF	0.0 (0.0)	1.00	0.0
		RV EDV	0.0 (0.0)	1.00	0.0
		RV ESV	0.0 (0.0)	1.00	0.0
		RV SV	0.0 (0.0)	1.00	0.0
		RV EF	0.0 (0.0)	1.00	0.0

Biventricular volumes and LV mass were indexed to body surface area. SD: standard deviation. ICC: intraclass correlation coefficient. CoV: coefficient of variation. LV: left ventricular. RV: right ventricular. EDV/ESV end-diastolic/systolic volume. SV: stroke volume. EF: ejection fraction

## Discussion

The present study demonstrates the feasibility of fully automated quantification of biventricular morphology and function and reveals its current pitfalls and limitations in a ‘real-world’ clinical setting. Several notable findings should be considered. First, automatically and manually derived volumes agree well in case of good image quality; however, severe differences occur in case of reduced image quality. Second, agreement is better for LV than for RV volumes. Third, demanding anatomical circumstances (e.g. in patients with repaired ToF) result in lower agreement. Forth, different field strengths or the presence of valve replacements do not impede automated assessments as long as image quality is preserved.

### Agreement of automated and manual assessment

CMR represents the reference standard for cardiac volume assessment [1] with incremental accuracy and reproducibility as compared to echocardiography [18]. However, CMR acquisition time is long and further requires time-consuming post-processing to extract clinically relevant information. Thus, efforts have been directed towards automated post-processing analyses based on deep-learning algorithms within the last decade [8, 19, 20]. The current literature demonstrates excellent agreement for automated and manual LV volume assessments [6, 10]; however, studies concerned with automatic RV segmentation are scarce [21]. Noteworthy, the study by Queirόs et al. [10] applied an automatic algorithm on cropped data, that is after manually defining the most basal and apical slices with subsequent cropping the SAX stack to include images effectively covering the LV before applying the automatic algorithm. Furthermore, ED and ES time points were manually pre-selected. However, the correct definition of the most basal slice is amongst the most challenging steps in SAX volume assessments and one of the most important source of observer variability [22], therefore representing a clear bias in testing the reliability of an automated algorithm. In the present study, we sought to simulate a real-world clinical scenario by randomly selecting patients from clinical routine imaging. We applied a commercially available automatic algorithm on clinically acquired SAX stacks – occasionally comprising both atria and ventricles – without any manual pre- or post-processing. The final data was acquired on 1.5 and 3.0 T scanners. Our data elaborates on the excellent agreement between automatically and manually derived volumes in case of good image quality, with overall better agreement for LV than for RV measurements. Indeed, quantification of RV volumes is generally more challenging as opposed to LV volumes due to the complex RV anatomy [23, 24]. Nevertheless, LV and RV stroke volumes were consistent in automated analyses in this patient group without intra- or extracardiac shunt.

Manual post-processing time took on average more than 11 min as compared to fully automated assessments with < 1 min. Importantly, automatic analyses of several CMR examinations (in this case 300 scans) run completely user-independent and were performed overnight. Furthermore, automated analyses promise to overcome limitations in observer variability, since the algorithm yields exactly the same measures when being reapplied by different users. Thus, the automated frame-work provides a highly reproducible approach and is able to extremely shorten post-processing times of CMR examinations with subsequent potential to improve cost-effectiveness [25]. Furthermore, the framework may provide ‘on-the-fly’ post-processing parallel to finishing the CMR scan (e.g. during late gadolinium enhancement acquisitions).

### Impact of image quality

Our data demonstrate that image quality is the leading determinant of accuracy for fully automatic volume assessment. In case of good image quality (image quality score of ≤1 adopted to the criteria proposed by Klinke et al. [13], Table 1), the bias of both LV and RV function was within acceptable limits. However, in case of reduced image quality (image quality score ≥ 2), a large bias of > 5% was observed for both LV and RV EF with wide LOA, particularly for RV EF. Importantly, the relevance of RV function and volumes is increasingly recognized in various diseases [26]. For example, the diagnosis of arrhythmogenic right ventricular cardiomyopathy is challenging and heavily relies on the assessment of RV EDV and RV EF [27]. If considered for clinical use and decision making, a precise volume assessment is of utmost importance, and cannot be achieved with the proposed fully automatic algorithm in case of impaired image quality yet.

### Technical and anatomical considerations

To further elucidate limitations of the commercially available software, we compared the agreement of automated and manually derived volumes for subgroups according to field strengths, the presence of aortic valve replacements as well as repaired ToF. Agreement was better at 1.5 T compared to 3 T scans; however, at 3 T considerably more artefacts (mainly due to inadequate breath-holding and shimming) were present. Reduced agreement at 3 T is therefore more likely a result of lower image quality. Due to the growing number of percutaneously implanted aortic valves [28] and increasing indications for CMR imaging [29] including aortic valve stenosis [30], the presence of valve replacement in CMR studies is likely to grow. As long as image quality was preserved in these patients, agreement of LV volumes remained acceptable, enabling the use of automated algorithms in this group of patients. In contrast, in patients with repaired ToF, both RV and LV agreement were considerably decreased, despite low image quality solely affecting the RV (metal artefacts resulting from sternal wires). Since LV image quality was good, reduced agreement is most likely due to the more demanding anatomy in these patients (distinctly larger RV than LV volumes), which points out the current limitations of fully automated analysis. Here, it remains to be investigated whether or not the proposed automatic deep-learning frame-work is able to further learn from these cases with subsequent improvement of accuracy.

### Limitations

The study’s conclusions are derived from the comparison of 300 automatically and manually quantified clinical CMR examinations from a single centre. Although manual contouring was performed by experienced observers, intra- and inter-observer variability may limit its use as a reference standard. Details of the automatic algorithm are not disclosed by the software vendor and therefore cannot be reported. RV mass was not measured, since the automatic algorithm does not provide RV mass quantification.

## Conclusion

Fully automated quantification of biventricular morphology and function is feasible and accurate in the majority of cases in a clinical routine setting and has the potential to extremely accelerate post-processing times and to improve reproducibility. However, in case of limited image quality or in patients with demanding anatomy (e.g. in patients with repaired ToF) the proposed fully-automatic frame-work does not yet provide satisfying results and still requires manual re-contouring.

## Additional files

Additional file 1: A. Myocarditis. B. TAVR. C. HCM. D. DCM. E. Repaired ToF. F. PAH. (ZIP 23844 kb)

Additional file 2 **Table S1.** Individual comparison between manual consensus and fully automated LV parameters based on the SCMR consensus data. **Table S2.** Agreement between expert manual and automated segmentation based on the SCMR consensus data. **Figure S1.** Agreement of automatically and manually derived consensus LV parameters based on the SCMR consensus data. (DOCX 144 kb)

## Abbreviations

AVR: Aortic valve replacement
bSSFP: Balanced steady state free precession
CI: Confidence interval
CMR: Cardiovascular magnetic resonance
CoV: Coefficient of variation
ECG: Electrocardiogram
EDV: End-diastolic volume
EF: Ejection fraction
ESV: End-systolic volume
ICC: Interclass correlation coefficient
IQR: Interquartile range
LV: Left ventricle/left ventricular
PAH: Pulmonary artery hypertension
RV: Right ventricle/right ventricular
SAX: Short axis
SCMR: Society for Cardiovascular magnetic Resonance
SV: Stroke volume
ToF: Tetralogy of Fallot
