# Supplementary material for: Fully automated quantification of biventricular volumes and function in cardiovascular magnetic resonance: applicability to clinical routine settings
Source: J Cardiovasc Magn Reson. 2019 Apr 25;21:24. doi: 10.1186/s12968-019-0532-9 (PMC8059518; doi:10.1186/s12968-019-0532-9)
Supplement: Supplementary file 2 — Table S1. Individual comparison between manual consensus and fully automated LV parameters based on the SCMR consensus data. Table S2. Agreement between expert manual and automated segmentation based on the SCMR consensus data. Figure S1. Agreement of automatically and manually derived consensus LV parameters based on the SCMR consensus data. (DOCX 144 kb) [file 12968_2019_532_MOESM2_ESM.docx]

**Additional file 2**

| **Table S1 Individual comparison between manual consensus and fully automated LV parameters based on the SCMR consensus data.** | | | | | | | | | |
| --- | --- | --- | --- | --- | --- | --- | --- | --- | --- |
|  |  | **EDV (ml)** | | **ESV (ml)** | | **LV Mass (g)** | | **LV EF (%)** | |
| Case | Pathology | Consensus | Automatic | Consensus | Automatic | Consensus | Automatic | Consensus | Automatic |
| 1 | Healthy | 104 | 109 | 48 | 46 | 70 | 70 | 54 | 58 |
| 2 | Infarct | 285 | 291 | 187 | 190 | 155 | 134 | 35 | 35 |
| 3 | Heart Failure | 293 | 300 | 254 | 230 | 134 | 143 | 13 | 23 |
| 4 | Infarct | 191 | 188 | 112 | 107 | 114 | 106 | 41 | 43 |
| 5 | Heart Failure | 369 | 345 | 268 | 253 | 171 | 153 | 28 | 27 |
| 6 | Hypertrophy | 151 | 155 | 48 | 58 | 175 | 163 | 68 | 63 |
| 7 | Infarct | 191 | 188 | 105 | 101 | 114 | 106 | 45 | 46 |
| 8 | Infarct | 201 | 205 | 142 | 144 | 122 | 119 | 30 | 30 |
| 9 | Infarct | 265 | 260 | 161 | 154 | 130 | 116 | 39 | 41 |
| 10 | Healthy | 158 | 152 | 65 | 56 | 133 | 146 | 59 | 63 |
| 11 | Healthy | 159 | 164 | 69 | 71 | 100 | 92 | 57 | 57 |
| 12 | Healthy | 222 | 223 | 88 | 88 | 122 | 107 | 60 | 61 |
| 13 | Infarct | 217 | 214 | 106 | 102 | 129 | 136 | 51 | 52 |
| 14 | Healthy | 170 | 163 | 74 | 77 | 117 | 105 | 56 | 53 |
| 15 | Hypertrophy | 167 | 201 | 76 | 52 | 193 | 172 | 54 | 74 |
| LV = left ventricle; EDV/ESV = end-diastolic/systolic volume, EF = ejection fraction. Note that LV mass was calculated from the end-diastolic frame. | | | | | | | | | |

| **Table S2** Agreement between expert manual and automated segmentation based on the SCMR consensus data. | | | | |
| --- | --- | --- | --- | --- |
|  | **Parameter** | **Mean Difference  (SD of the Diff.)** | **ICC (95% CI)** | **CoV (%)** |
| **SCMR Consensus Data** | LV Mass | 7.3 (10.6) | 0.95 (0.80-0.99) | 8.3 |
| **(n=15)** | LV EDV | -1.1 (12.0) | 0.99 (0.98-1.00) | 5.7 |
|  | LV ESV | 4.9 (9.5) | 0.99 (0.98-1.00) | 8.1 |
|  | LV EF | -2.4 (5.8) | 0.96 (0.87-0.99) | 12.2 |
| SD: standard deviation, ICC: intraclass correlation coefficient, CoV: coefficient of variation, LV: left ventricular, EDV/ESV end-diastolic/systolic volume, EF: ejection fraction | | | | |

**Figure S1 Agreement of automatically and manually derived consensus LV parameters based on the SCMR consensus data.**

Bland Altman plots (automatic – manual) are shown for LV parameters (15 subjects). LV: left ventricle, EDV/ESV: end-diastolic/systolic volume, EF: ejection fraction, Δ: difference.
